# Novel Homozygous Variants in CIDEC and WRN in a Young Female with Lipodystrophy and Thyroid Cancer

**DOI:** 10.3390/ijms27020646

**Published:** 2026-01-08

**Authors:** Nivedita Patni, Chao Xing, Chun-Yuan Huang, Rebecca J. Brown, Abhimanyu Garg

**Affiliations:** 1 Division of Endocrinology, Department of Pediatrics, UT Southwestern Medical Center, Dallas, TX 75390, USA; nivedita.patni@utsouthwestern.edu; 2 McDermott Center for Human Growth and Development, UT Southwestern Medical Center, Dallas, TX 75390, USA; chao.xing@utsouthwestern.edu (C.X.); chunyuanh@mail.smu.edu (C.-Y.H.); 3 Lyda Hill Department of Bioinformatics, UT Southwestern Medical Center, Dallas, TX 75390, USA; 4 O’Donnell School of Public Health, UT Southwestern Medical Center, Dallas, TX 75390, USA; 5 National Institute of Diabetes and Digestive and Kidney Diseases, National Institutes of Health, Bethesda, MD 20892, USA; brownrebecca@niddk.nih.gov; 6 The Section of Nutrition and Metabolic Diseases, Division of Endocrinology, Department of Internal Medicine and the Center for Human Nutrition, UT Southwestern Medical Center, Dallas, TX 75390, USA

**Keywords:** partial lipodystrophy, papillary thyroid carcinoma, *WRN*, *CIDEC*, FSP27

## Abstract

Autosomal recessive familial partial lipodystrophy type 5 (FPLD5) due to a homozygous NP_001186481.1; p.E186* *CIDEC* variant has previously been reported in a 19-year-old female with diabetes mellitus, hypertriglyceridemia, and hepatic steatosis. Now, we report an 18-year-old Hispanic female who presented with FPL, along with hirsutism, acanthosis nigricans, and marked insulin resistance, and was found to have an extremely rare homozygous variant in *CIDEC* (NM_001199623.2:c.224G>T; NP_001186552.1; p.Ser75Ile) by whole exome sequencing. She also harbored a novel homozygous variant in *WRN* (NM_000553.4:c.1856T>G; NP_000544; p.Leu619Arg). Both serine 75 of the CIDEC protein and leucine 619 of the WRN protein were well conserved across species. She developed an invasive papillary thyroid carcinoma at the age of 17 years. Our report confirms the previously reported association of the biallelic *CIDEC* variant with the FPL phenotype and also highlights the extremely rare possibility of co-occurrence of FPLD5 with thyroid cancer, a clinical feature of Werner syndrome. Thus, our patient may not only need surveillance for the metabolic complications of FPLD5, such as diabetes, hypertriglyceridemia, and hepatic steatosis, but also for WRN-associated neoplasms and features of premature aging.

## 1. Introduction

Autosomal recessive familial partial lipodystrophy (FPL) syndromes due to biallelic variants in the *CIDEC*, *LIPE*, and *PCYT1A* genes have only been reported in a small number of patients [1,2,3,4]. For example, only one 19-year-old Ecuadorian female with FPL harboring a biallelic homozygous null variant (NP_001186481.1; p.E186*) in Cell death-inducing DFFA-like effector C (CIDEC) has been reported previously [4]. She had childhood-onset acanthosis nigricans, hepatic steatosis, extreme hypertriglyceridemia, uncontrolled diabetes mellitus, and, on follow-up, developed microalbuminuria and hypertension [4]. However, all these phenotypic features of this patient could not be conclusively attributed to the *CIDEC* variant [4]. Here, we provide further evidence for CIDEC as the locus of autosomal–recessive FPL by reporting another young female harboring a homozygous *CIDEC* missense variant, who, interestingly, also had a novel homozygous variant in *WRN*.

## 2. Case Report

The study protocol was approved by the National Institute of Diabetes and Digestive and Kidney Diseases, National Institutes of Health, Bethesda, Maryland, USA. The patient and her mother provided written informed consent, including for the use of the patient’s photographs, and the record of informed consent has been retained.

This 18-year-old Hispanic female of Ecuadorian descent had thelarche at the age of 9 years and menarche at 13 years, when a gradual decrease in subcutaneous fat in her extremities was noticed. She also had hirsutism but had mostly regular menstrual cycles. The patient’s serum testosterone was elevated (157 ng/dL, normal range 14 to 53 ng/dL) at age 12 years.

**Figure 1:**
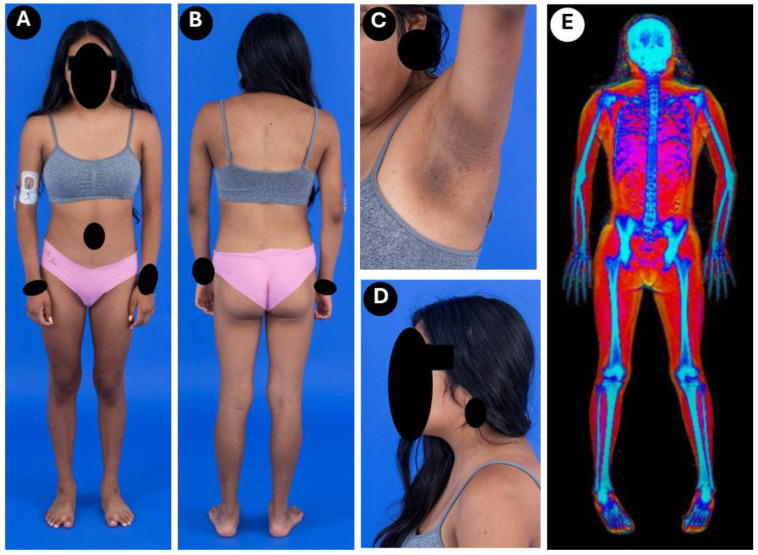
Phenotypic features and Dual-energy X-ray absorptiometry (DEXA) scan of the patient at the age of 15 years. (**A**) Anterior and (**B**) posterior view of the patient. Note decreased subcutaneous (sc) fat in the forearms, hips, and lower extremities, and preserved sc fat around the shoulders and upper arms. (**C**) Severe acanthosis nigricans in the axilla. (**D**) Increased adiposity in the neck and a small dorsocervical fat pad. (**E**) DEXA scan showing preserved fat around the shoulders and upper arms; however, sc fat in the forearms and lower extremities is markedly reduced. Total body fat by DEXA was 26.8% (−0.8 SD); regional fat in the upper extremities was 28.75% (−0.65 SD), and in the lower extremities was 24.1% (−2.3 SD). Her bone density measurements for the total whole body were 0.990 (z score 0.2), for the Lumbar region (L1 to L4) were 0.914 (z score 0.4), left femoral neck 0.789 (z score −0.1), total left hip 0.825 (z score −0.5), and at the left forearm were 0.64 (z score 0.5).

Her height at age 18 years was 1.55 m, and her weight was 50.9 kg with a body mass index of 21.2 kg/m^2^ (49th percentile). Her father’s height was 1.80 m, and her mother’s was 1.52 m, with a mid-parental height of 1.60 m. Her height was within 2SD of the mid-parental height. She had increased adiposity in the face and a small dorsocervical fat pad (Figure 1). Subcutaneous fat in the forearms and the lower extremities was markedly decreased, and minimal gluteal fat was present. Acanthosis nigricans was present in the neck and axillary regions. She had increased muscularity of the lower extremities and some phlebomegaly. The patient reported normal exercise tolerance.

**Figure 2:**
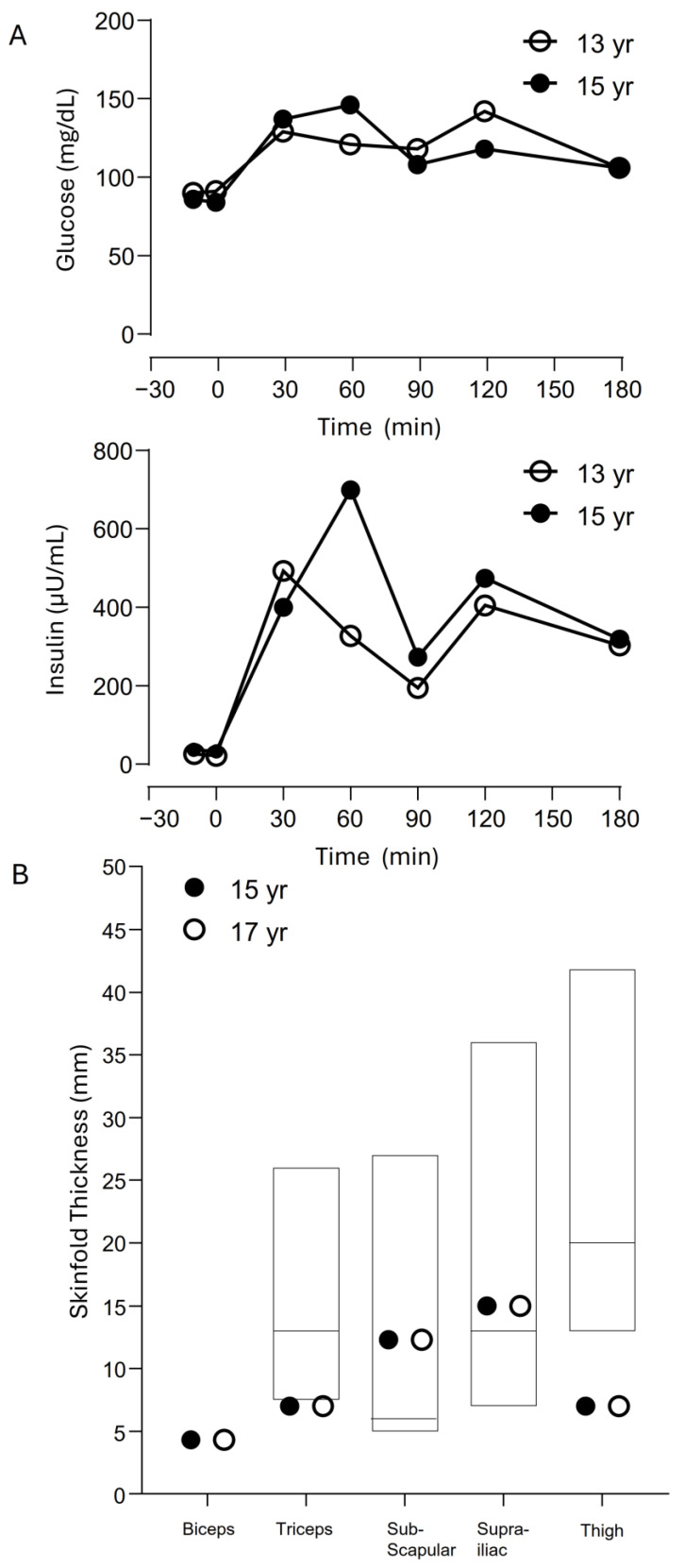
Oral glucose tolerance test and skinfold thickness measurements of the patient. (**A**) Oral Glucose tolerance test (OGTT) results at ages 13 and 15 years. At age 13 (unfilled circles), the OGTT showed normal fasting glucose (91 mg/dL) with modestly elevated fasting insulin (21.8 µU/mL), a mild glucose rise post-load, and marked hyperinsulinemia peaking at 493.3 µU/mL at 30 min and remaining elevated at 404.9 µU/mL at 120 min. By age 15 (solid circles), fasting glucose levels remained normal (84 mg/dL), but fasting insulin levels were higher (32.7 µU/mL). Additionally, post-load 2 h plasma glucose peak was higher (146 mg/dL vs. 129 mg/dL). Post-glucose insulin response was more exaggerated and prolonged, peaking at 699 µU/mL at 60 min, with persistently elevated levels throughout the test, indicating worsening insulin resistance. Oral glucose was administered at time 0 min. (**B**) Skinfold thickness at 15 years (solid circles) and 17 years (unfilled circles). The bars show 10th to 90th percentile values of normal age-matched girls [5], with the median value marked by a horizontal line. At age 15, biceps, triceps, subscapular, suprailiac, and thigh skinfold thicknesses were 4.7 mm, 5.0 mm, 12.0 mm, 10.0 mm, and 6.0 mm, respectively. At age 17, the corresponding values were 4.3 mm, 7.0 mm, 12.3 mm, 15.0 mm, and 7.0 mm, respectively. These graphs show markedly reduced values in both the upper and lower extremities, and at the triceps and thigh were below the 10th percentile of normal controls, while the sub-scapular and supra-iliac skinfold thickness were above the 50th percentiles.

Skinfold thickness measurements at age 15 years revealed markedly reduced values in both the upper and lower extremities, especially at the triceps and thigh, which were below the 10th percentile of normal controls (Figure 2) [5]. Regional fat by dual energy X-ray absorptiometry was normal in the upper extremities (28.75%, −0.65 SD) but was reduced in the lower extremities (24.1%, −2.3 SD) (Figure 1E) [6]. Her bone density measurements were normal (Figure 1E).

Her serum total cholesterol was 141 mg/dL; triglycerides, 60 mg/dL; high-density lipoprotein cholesterol, 41 mg/dL; and blood hemoglobin A1c, 4.8%. Oral glucose tolerance tests at the ages of 13 and 15 years revealed impaired glucose tolerance and marked hyperinsulinemia (Figure 2). At age 13 years, her serum aspartate aminotransferase was 12 U/L (normal 17–33); alanine aminotransferase (ALT), 17 U/L (normal 5–30); uric acid, 3.6 mg/dL (normal 2.2–6.4); and leptin (ELISA, Millipore) was 16.4 ng/mL. Abdominal ultrasound at the age of 15 years revealed normal liver size (13.8 cm), with normal stiffness (elastography 1.53 m/s) [7] and echogenicity, and normal spleen size (181 mL).

Routine screening neck ultrasound at age 13 years showed a 5 mm × 4 mm × 4 mm right thyroid lobe nodule, which by age 17 years had increased in size to 14 mm. The nodule was non-encapsulated with infiltration of the right lower lobe and multiple punctate echogenic foci consistent with microcalcifications. Needle biopsy of the thyroid nodule showed papillary thyroid carcinoma. The patient underwent a total thyroidectomy with right central neck dissection, and oral levothyroxine was initiated. The patient was treated with radioactive iodine ablation after the surgery, and subsequent imaging at age 18 years showed no evidence of residual tumor. She had no cataracts, premature graying, loss of hair, or sclerodermatous skin changes.

### Genome Sequencing and Analysis

Genomic DNA was isolated from peripheral blood using the Easy-DNA kit (Invitrogen, Carlsbad, CA, USA). The proband (FPL361.3) and her mother (FPL361.1) underwent whole exome sequencing (WES) using the Integrated DNA Technologies xGen Exome Research Panel V.1.0 on the Illumina platform. The mean coverage of the targeted regions was >50-fold, with >80% of bases covered by >20-fold reads in both samples. Sequences were aligned to the human reference genome b37, and variants were called using the Genome Analysis Toolkit (v3.8) and annotated using SnpEff (v5.1).

The inbreeding coefficients were estimated to be 0.087 and 0.118 for FPL361.1 and FPL361.3, respectively, which suggested consanguinity within the family. Therefore, we first filtered for variants that were homozygous in the proband but heterozygous in her mother under a recessive model. In the meantime, we performed runs of homozygosity (ROH) analysis searching for segments greater than 1 Mb in the proband but not in her mother using BCFtools/ROH [8] and copy number variation (CNV) detection using CNVkit [9] comparing FPL361.3 vs. FPL361.1. In addition, we filtered for variants that were heterozygous in the proband and absent in her mother under a dominant model with special attention to lipodystrophy candidate genes, including *AGPAT2*, *AKT2*, *BSCL2*, *CAV1*, *CIDEC*, *EPHX1*, *LIPE*, *LMNA*, *MTX2*, *NOTCH3*, *PCYT1A*, *PIK3R1*, *PLAAT3*, *PLIN1*, *POLD1*, *PPARG*, *PSMB8*, *PTRF*, *TYMP*, and *ZMPSTE24*. We filtered for rare missense, nonsense, splicing, or frameshift variants meeting the following criteria: minor allele frequencies (MAF) less than 0.001 in each of the subpopulations in the genome aggregation database (gnomAD v4.1.0; http://gnomad.broadinstitute.org/, accessed on 26 June 2025), a genomic evolutionary rate profiling++ score [10] greater than 2.0, and a combined annotation-dependent depletion score [11] greater than 15. As the father’s DNA was unavailable, de novo variants were not considered.

**Figure 3:**
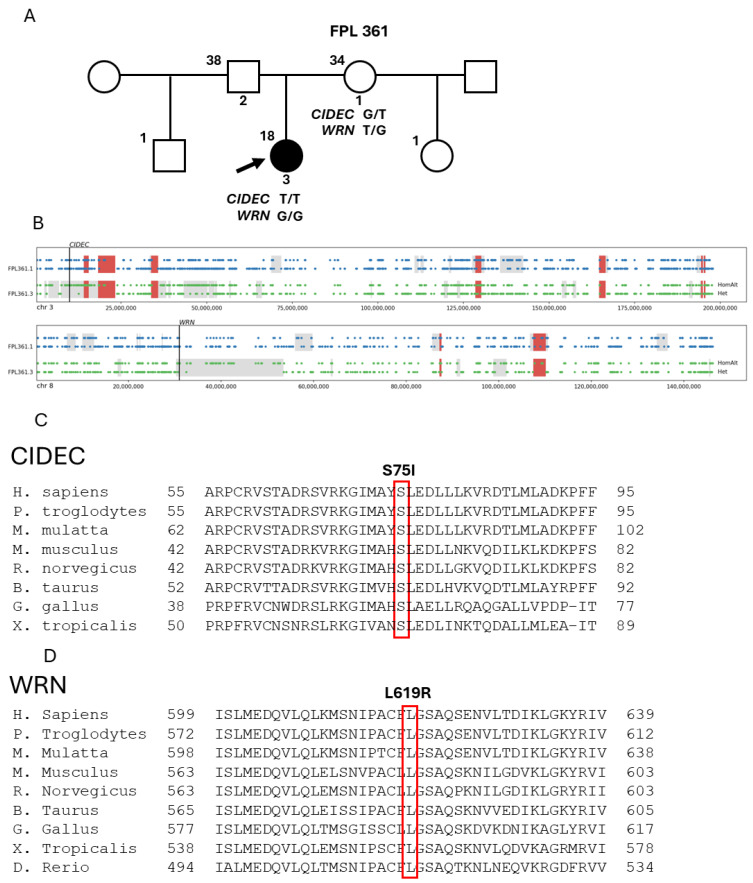
FPL361 Pedigree, regions of homozygosity on chromosomes 3 and 8 encompassing the disease-causing variants in *CIDEC* and *WRN*, and conservation of the mutated residues across the species. (**A**) FPL361 Pedigree. Circles denote females, and squares denote males, and the numbers on the top left of the symbols denote current age in years, and under the symbols denote pedigree number. The slanted arrow indicates the proband, who is shown as a filled black symbol, while the unaffected subjects are shown as unfilled symbols. The patient’s mother, 34 years old (1.52 m, 62.7 kg), and father, 38 years old (1.80 m, 94.1 kg), were both healthy. The genotypes of the proband and her mother are given under the symbols. For *CIDEC*, T/T indicates homozygous c.224G>T variant and G/T indicates heterozygous variant; and for *WRN*, G/G indicates homozygous c.1856T>G variant and T/G indicates heterozygous variant. The minor allele frequency (MAF) of c.224G>T variant in *CIDEC* is 2.0 × 10^−5^ in gnomAD, and the *WRN* variant is absent. Both variants are located at highly conserved sites—GERP++ scores equal to 5.3 and 5.5, respectively, and are predicted to be of high impact—CADD scores equal to 23.2 and 26.9, respectively. (**B**) Schematic of segments on chromosomes 3 and 8 of the proband (FPL361.3) and her mother (FPL361.1), based on GRCh37/hg19 coordinates. For each individual, the top line displays markers with homozygous genotypes and the bottom line displays markers with heterozygous genotypes. The inferred homozygous regions are highlighted in color blocks: red for regions common to both and gray for regions unique to one individual. The *CIDEC* and *WRN* loci are indicated. (**C**) Multiple species alignment of the region surrounding Ser75 of human CIDEC and (**D**) multiple species alignment of the region surrounding Leu619 of human WRN performed in the COBALT multiple alignment tool (https://www.ncbi.nlm.nih.gov/tools/cobalt/cobalt.cgi, accessed on 26 June 2025). CIDEC Ser75 and WRN Leu619 are conserved across all the species. The Genbank accession numbers are: Homo sapiens NP_006102.2, Mus musculus NP_803421.1, Rattus Norvegicus NP_569117.2, Danio rerio NP_998217.1, Box taurus NP_001030419.1, Xenopus tropicalis XP_012820301.1, Gallus gallus NP_001006571.2, Macaca mulatta NP_001253662.1, Pan troglodytes NP_001253662.1.

WES analysis revealed that the proband carried one extremely rare homozygous missense variant: chr3, g.9918772C>A (rs766353274; NM_001199623.2: c.224G>T; NP_001186552.1: p.Ser75Ile) in *CIDEC*, and one novel homozygous missense variant: chr8, g.30949372T>G (NM_000553.4: c.1856T>G; NP_000544: p.Leu619Arg) in *WRN* (Figure 3A). The proband, but not her mother, had regions of homozygosity (ROH) including 6.7 Mb on chromosome 3 and 23.1 Mb on chromosome 8, where the variants were located (Figure 3B). Both serine 75 of the CIDEC protein and leucine 619 of the WRN protein were well conserved across species (Figure 3C,D). The mother was a heterozygous carrier of both variants. There was no heterozygous pathogenic variant or CNVs identified in lipodystrophy candidate genes in the proband.

The minor allele frequency of the *CIDEC* p.Ser75Ile variant is 2.0 × 10^−5^ in gnomAD, 1.8 × 10^−5^ in All of US, and 1.7 × 10^−5^ in UK Biobank, without any homozygous carrier observed; the *WRN* p.Leu619Arg variant is absent in all these databases. Both variants are located at highly conserved sites—GERP++ scores equal to 5.3 and 5.5, respectively, and are predicted to be of high impact—CADD scores equal to 23.2 and 26.9, respectively. However, both variants would be classified as VUS (variant of unknown significance) by the ACMG criteria [12].

## 3. Discussion

CIDEC is a crucial lipid droplet protein that promotes lipid droplet formation and fusion in adipocytes [13]. It is predominantly expressed in white adipose tissue and plays a key role in triglyceride storage and negatively regulates lipolysis [14]. Only a single patient with a homozygous null variant in CIDEC has been reported to be associated with FPL type 5 (FPLD5), characterized by peripheral fat loss from the limbs and gluteal region with normal to increased central fat deposition in the face and trunk, and severe insulin resistance [4]. Our report provides further confirmation of the association of FPL phenotype with the CIDEC variants. Similarly to the previously reported patient with FPLD5, our patient also had a loss of fat from the extremities with excess fat in the face and upper back. Interestingly, our patient did not have any hepatic steatosis or hepatomegaly and also had normal serum triglyceride levels, in contrast to the previously reported patient who had marked hepatic steatosis, extreme hypertriglyceridemia, and acute pancreatitis. However, the metabolic deterioration in the previously reported patient might have been due to uncontrolled diabetes mellitus (hemoglobin A1c 11.0% to 16.3%) despite taking 1.6 units of insulin per kg body weight subcutaneously daily. Our patient also had normal serum leptin levels, whereas the previously reported patient had low levels of serum leptin. However, our patient also had severe insulin resistance.

We further expand the scope of phenotypic findings of FPLD5 due to CIDEC variants. For example, our patient had normal bone density and exercise tolerance in contrast to the findings of osteoporosis and myopathy in the Cidec, also called fat-specific protein 27 (Fsp27), knock-out mouse model [14]. Further follow-up of both patients will be needed to see if they develop muscle or bone pathology later in life. While Fsp27-null mice are lean and resistant to diet-induced obesity and insulin resistance [15,16], under energetic stress, they display hepatic steatosis and systemic insulin resistance [17]. Fsp27 knock-out mice exhibit multilocular lipid droplets in white adipocytes [15,16]; similar findings were observed in the previously reported patient with FPLD5, including a mixed population of uni- and multi-locular white adipocytes from axillary subcutaneous adipose tissue biopsy [4].

The CIDEC p.Ser75Ile variant identified in our patient is located in a highly conserved region of the protein. While this specific variant has been reported in the heterozygous state in population databases, no homozygotes have been previously documented. It is possible that our patient with a missense variant, p.Ser75Ile, had less severe lipodystrophy compared to the previously reported patient, who had a null variant, p.Glu186*; however, no skinfold thickness or regional body fat data from DEXA scans were available for the previously reported patient [4]. Published magnetic resonance images of the previously reported patient did show a marked loss of subcutaneous fat from both the upper and lower extremities, with increased fat accumulation in the neck and intra-abdominal region [4]. Interestingly, neither patient had increased subcutaneous fat accumulation in the pubic region and labia majora, which is seen in females with FPL, Dunnigan, due to *LMNA* variants [18].

Interestingly, our patient also had a missense homozygous variant in *WRN*. *WRN* encodes a RecQ DNA helicase involved in DNA replication and repair, and telomere maintenance [19]. Biallelic loss-of-function pathogenic variants in *WRN* cause Werner syndrome, a rare autosomal recessive disorder characterized by premature aging, with the following four cardinal signs: bilateral cataracts, premature graying or thinning of scalp hair, sclerodermatous skin changes, and short stature. In addition, patients with Werner syndrome also present with thin limbs, pinched facial features, voice change, hypogonadism, type 2 diabetes mellitus, soft tissue calcification, neoplasm(s), ulcers on legs, and atherosclerosis [20,21,22,23,24]. Although our patient did not have cataracts, facial features of premature aging, short stature, sclerodermatous skin, or osteoporosis, she did develop papillary thyroid carcinoma at a very young age, which provides supportive evidence for the pathogenicity of the novel homozygous missense variant, p.Leu619Arg, in WRN. This variant affects the protein’s helicase domain, which is critical for its function [25]. WRN deficiency leads to genomic instability and can increase the risk of neoplasms, particularly sarcomas, meningiomas, thyroid cancer, and melanoma [26,27,28,29].

Most of the pathogenic variants causing Werner syndrome are loss-of-function variants (null, frameshift, or indels), and only six other missense variants have been reported so far [19,30,31,32,33,34]. However, three of them were in trans with another null variant (p.Arg637Trp with p.Val1082fsX [31]; p.Gly574Arg with p.I662fsX [34]; and p.Met1350Arg with p.N1197fsX [32]), and another patient had double missense homozygous variants (p.Lys125Asn with p.Lys135Glu) [19]. Recently, Sezer et al. [33] reported a consanguineous family with a homozygous, novel *WRN* missense variant p.Trp200Cys in four affected siblings (13–23 year-old males) with interstitial lung disease, spontaneous pneumothorax, and progressive pulmonary failure. However, none of the affected subjects (three of whom died at ages 16–23 years) had any other cardinal signs of Werner syndrome besides growth failure. The lung phenotype is extremely rare in confirmed patients with Werner syndrome [35]. No functional studies were conducted to ascertain the pathogenicity of the *WRN* variant. Interestingly, all affected siblings also harbored a c.357+1G>T homozygous variant in *SFXN5*. There are no previous reports of human phenotypes associated with homozygous null variants of *SFXN5*. Therefore, whether the phenotype was due to atypical Werner syndrome, a new clinical entity associated with the *WRN* variant, or due to the *SFXN5* variant was not clear [33]. Overall, which clinical features of Werner syndrome may be caused by exclusively homozygous missense rare variants can not be ascertained from these reports.

While classical lipodystrophy is not a recognized feature of Werner syndrome, patients often display a characteristic body habitus with thin limbs and relatively preserved truncal fat, resembling a partial lipodystrophy phenotype [36]. Recent studies have suggested that WRN may play a role in adipocyte differentiation and function, potentially linking it to lipodystrophy syndromes [37,38]. Thus, it is possible that the homozygous *WRN* variant in our patient may have contributed to her lipodystrophic phenotype and insulin resistance.

## 4. Conclusions

In conclusion, our report confirms the previously reported association of a biallelic CIDEC variant with familial partial lipodystrophy phenotype. Our case also highlights the extremely rare possibility of co-occurrence of FPLD5 with thyroid cancer, a clinical feature of Werner syndrome. Thus, in future, our patient may not only need surveillance for metabolic complications of FPLD5 such as diabetes, hypertriglyceridemia, and hepatic steatosis, but also for WRN-associated neoplasms and features of premature aging.

## Data Availability

The individual-level whole-exome sequencing data sets cannot be released because of the Health Insurance Portability and Accountability Act and Genetic Information Nondiscrimination Act regulations to protect patients’ genetic privacy.
